# Past and Future Considerations for Heavy-Duty Diesel Engine Emissions

**DOI:** 10.1289/ehp.112-a727

**Published:** 2004-09

**Authors:** Allen R. Schaeffer

**Affiliations:** Diesel Technology Forum, Frederick, Maryland, E-mail: aschaeffer@dieselforum.org

In her Commentary in the June issue of *EHP*, “Diesel Exhaust: A Moving Target,” Janet Arey (2004) makes a strong point for standardizing diesel exhaust reference material for future research due to the changes in diesel technology and resulting emissions.

Arey (2004) appropriately recognized that diesel exhaust particulates are a small category of emissions in what is truly a complex mixture of ambient particles. According to the U.S. Environmental Protection Agency’s (EPA’s) most recent emissions inventory (U.S. EPA 2003), emissions from all diesel sources (on-road light and heavy-duty, off-road, marine and rail) in 2001 accounted for 4.37% of the nation’s fine particle inventory.

Recognizing the shift toward dramatically lower emissions and potential changes in the composition of those emissions, diesel engine manufacturers have initiated a unique broad-ranging stakeholder project known as the Advanced Collaborative Emissions Study, or ACES (French 2003). The objective of this collaborative government, academic, and industry research program is to develop the necessary data to assess and characterize in a timely manner (i.e., in the 2007–2008 timeframe) the emissions and any potential health effects from real-world exposures to exhaust from advanced prototype 2007–2010 heavy-duty engines, after-treatment systems, and reformulated fuels. This effort includes 794 emissions characterizations, chronic animal exposure studies, and short-term studies on allergic responses, and it is expected to publish findings in 2009–2010 (Warren 2004).

In further noting Arey’s appeals on the “need to understand the atmospheric chemistry of diesel and other vehicle exhaust” (Arey 2004), alternative fuels should also be evaluated. Now, more than ever, this is of particular significance as the use of alternative-fueled vehicles has increased, but the understanding and research of these emissions in the atmosphere have not kept pace.

A number of recently published studies have assessed the emissions from alternative-fueled heavy-duty vehicles. In a study of school buses running on diesel and compressed natural gas (CNG), low-emitting clean diesel technology had the lowest level of both U.S. EPA regulated emissions and toxic air compounds as defined by the California Air Resources Board (Ullman et al. 2003).

Similarly, the California Air Resources Board conducted a small-scale research project comparing transit buses using CNG to buses using advanced clean diesel technology (cleaner diesel fuel and particulate traps) (Ayala et al. 2002). This limited study found that the clean diesel bus had fewer emissions of toxic compounds than the CNG bus, and that both types of buses (CNG and those using filters and cleaner fuel) were superior to conventional diesel fuel and engines (Holmean and Ayala 2002). Even though the CNG bus was not equipped with an oxidation catalyst, the higher emissions of air toxics (1,3-butadiene, formaldehyde, etc.) could be expected by similar technology configurations currently in use around the country. For some areas that have aggressively promoted the use of so-called clean alternative fuels such as CNG without a complete understanding of their emissions profiles, these findings call for an additional consideration by Arey and other atmospheric chemists.

As the subtitle on the cover of the June issue of *EHP* appropriately suggests, diesel technology is a moving target—moving rapidly toward very low emissions across the board in all engine types and categories, with clearly defined pathways and time frames. For highway vehicles, 2004 model heavy-duty diesel engines have only one-eighth the level of emissions of nitrogen oxides and particulate matter compared to those built a dozen years ago, with 90% in additional reductions of particulate matter on track beginning in 2007 (U.S. EPA 2001). The U.S. EPA recently issued final rules for the fourth round of new lower emissions standards for off-road machines and equipment in the farming, construction, and industrial sectors, along with proposed rules for cleaner fuel requirements for marine vessels and locomotives (U.S. EPA 2004). Taking effect beginning in 2008 and at full implementation in 2014, these standards will converge at virtually the same low levels as highway engines.

Finally, an additional consideration, which was not identified by Arey (2004) but is significant, is the future impacts of applying new reformulated lower-sulfur diesel fuels and emissions filters on existing engines and equipment of various ages and types, an effort increasingly under way at the state and federal levels. Given this level of rapid change, establishing standardized reference materials will be particularly challenging.
